# Evaluation of a hospital-based surveillance system for birth defects in Chennai, India

**DOI:** 10.18203/2394-6040.ijcmph20214293

**Published:** 2021

**Authors:** Anoop Velayudhan, Suresh Seshadri, Sujatha Jagadeesan, Jayanti Saravanan, Rajesh Yadav, Lorraine F. Yeung

**Affiliations:** 1 Epidemic Intelligence Service India Programme-National Centre for Disease Control, New Delhi, India; 2 Fetal Care Research Foundation, Chennai, Tamil Nadu, India; 3 CDC India, New Delhi, Delhi, India; 4 National Center on Birth Defects and Developmental Disabilities, Centers for Disease Control and Prevention, Atlanta, Georgia, USA

**Keywords:** Birth defects, Registries, India, Surveillance, Evaluation

## Abstract

The Birth Defects Registry of India-Chennai (BDRI-C) was created in 2001 to monitor birth defects and provide timely referrals. Using established guidelines to evaluate surveillance systems, we examined the following attributes of BDRI-C to help strengthen the registry: simplicity, flexibility, data quality, representativeness, acceptability, timeliness, and stability. We reviewed BDRI-C documents, including reporting forms; interviewed key informants; and calculated data completeness, coverage, and reporting time. BDRI-C captured 14% of the births in Chennai April 2013 - March 2014. About 7% of institutions in Chennai registered in BDRI-C, and of those registered, 37% provided data in 2013. Median reporting time was 44 days after birth in 2013. BDRI-C is a useful, simple, flexible, and timely passive birth defects surveillance system; however, improvements can be made to ensure BDRI-C is representative of Chennai, data processing and quality checks are on-going, and the system is acceptable for member institutions and stable.

## INTRODUCTION

Birth defects or congenital anomalies are structural or functional malformations, including metabolic disorders, present at the time of birth.^1^ They are a leading cause of infant mortality and morbidity worldwide, affecting about 3-6% of all births and one in every five infant deaths.^2^ In India, the overall prevalence of birth defects is estimated at 64.3 per 1,000 live births.

Estimating birth defects prevalence in developing countries is difficult due to lack of robust surveillance systems and clinical information on birth defects.^3^ The Fetal Care Research Foundation recognized the gap and created the Birth Defects Registry of India (BDRI) in MediScan Systems in 2001 in Chennai, Tamil Nadu.^4^ The purpose of BDRI is to establish birth defect registries throughout India to monitor secular trends and clustering of birth defects, help identify defects early, and provide timely referrals. BDRI uses passive voluntary hospital-based surveillance to monitor structural or chromosomal anomalies found in a live birth, an intrauterine fetal demise, a stillbirth, or a medically terminated fetus from delivery through the first year of life.^5^ Private and public hospitals or clinics that conduct deliveries are invited to join BDRI after sensitization and training for standardized reporting. Initially, BDRI started with several hospitals in Chennai and has now expanded to 750 member institutions from 28 states and three union territories.

**Figure 1:**
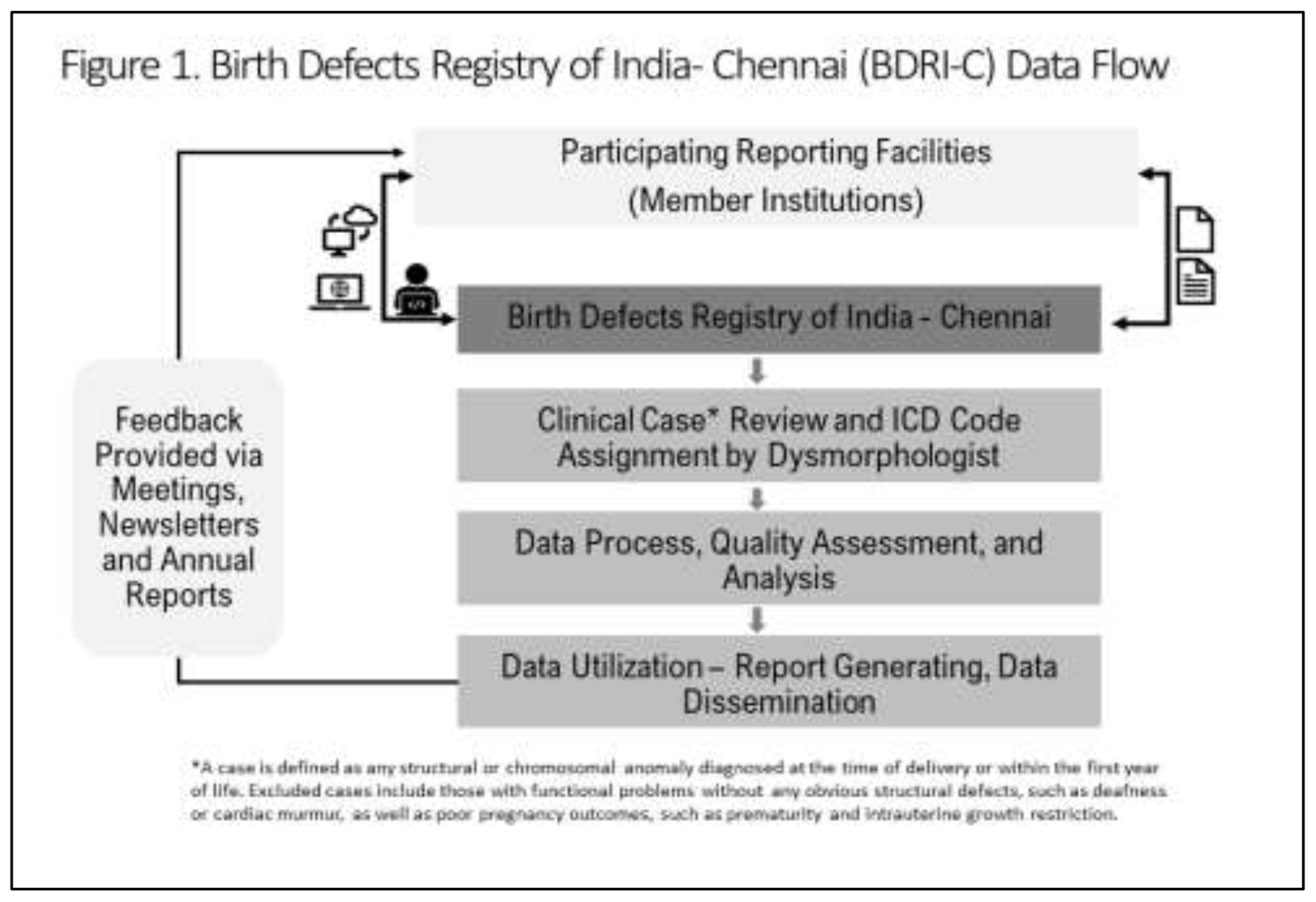
Birth defects registry of India-Chennai (BDRI-C) data flow.

BDRI in Chennai (BDRI-C) surveillance data flow, including processing, analyses, and reporting, as well as communication between BDRI-C and member institutions are shown in Figure 1.

We evaluated the BDRI-C to identify system performance and gaps as well as provide suggestions to help strengthen birth defects data collection and use for institutions in Chennai.

## METHODS

### Study design

We reviewed BDRI-C print materials, including reporting formats; report registers from April 2013-April 2014; and BDRI annual reports from 2001-2014. We also interviewed 11 key informants (doctors, data entry workers, and staff) using semi-structured interviews to collect data on selected attributes of BDRI-C. Data collected were coded by themes and analyzed. Finally, we calculated percentage of missing data for selected variables, representative coverage, and reporting time from representative institutions from both government and private sector institutions in Chennai.

### Study tools

We examined BDRI-C using the Centers for Disease Control and Prevention’s Updated Guidelines for Evaluating Public Health Surveillance Systems that covers the system attributes of simplicity, flexibility, data quality, representativeness, acceptability, sensitivity, predictive value positive, timeliness, and stability (Table 1).^5^

We assessed simplicity by considering BDRI-C data collection methodology and data dissemination. We examined flexibility as BDRI-C’s ability to modify reporting forms (i.e., add new fields). Data reporting forms were checked for completeness. We examined reporting records from 17 institutions for 2013; nine institutions provided paper reporting forms for 95 cases, and eight institutions provided 55 online reports. We defined representativeness as the proportion of participating institutions reporting data to BDRI-C and the percent of births covered by these participating institutions. We examined the proportion of institutions reporting data to BDRI-C from 2011-2013 and the births covered from April 2011 through March 2014. We assessed acceptability based on the willingness of persons and organizations to participate in the surveillance activities. We examined timeliness of online reporting by assessing the time from date of birth to date of reporting to BDRI-C. We assessed the system’s stability by examining the variations over time of the number of institutions participating in and reporting data to BDRI-C. No other reporting system for capturing birth defects existed in India in 2014; therefore, sensitivity and positive predictive value of the surveillance system could not be evaluated. This evaluation was done as part of the India Epidemic Intelligence Service Program, as requested by BDRI-C, for program improvement. Formal ethical approval was deemed not applicable.

## RESULTS

### Usefulness

BDRI-C is helpful in linking families impacted by birth defects to needed services; however, inconsistent reporting and varying data sources limit its ability to identify clusters and trends for birth defects in Chennai.

### System attributes

#### Simplicity

The reporting structure was relatively easy to understand by reporting member institutes, with bidirectional feedback between BDRI-C and reporting members (Figure 1). Functionally, the online reporting system was easier to use since the paper-based form required sending the forms from the reporting institutions via courier or staff to MediScan for entry into BDRI-C.

#### Flexibility

The BDRI-C reporting form was thrice changed to either reduce or add fields. It was initially reduced from 84 to 21 fields in 2007, then four new fields added in 2011, and another new field added in 2012 (Table 1). These changes were due to feedback from reporting members and BDRI-C joining the International Clearinghouse for Birth Defects Surveillance and Research (ICBDSR).

#### Data quality

Among 26 fields in the 150 reporting forms examined, contact information had the most missing data. Phone number was the most frequently missing variable (34%, 32/95 in paper forms and 31%, 17/55 in online reports), followed by incomplete addresses for the online reports (25%, 14/55) only.

#### Representativeness

Among 698 maternity institutions registered for birth in Chennai, only 46 (7%) institutions were registered in BDRI-C. Among registered institutions, 21 (46%), 14 (30%) and 17 (37%) reported data in 2011, 2012, and 2013, respectively (reporting institutions can vary by year). BDRI-C collected information on only 14% (11,763/81,209) of the births in Chennai from April 2013 to March 2014, which was a decrease in coverage from April 2011 to March 2012 (40%) and April 2012 to March 2013 (30%).

#### Acceptability

Very few maternity institutions in Chennai registered in BDRI-C and of those that registered, very few provided data in 2011-2013.

Staff turnovers at the reporting member institutions required on-going training and re-sensitization. Data review meetings in the institutions were perceived as academic and reported as being useful only for pediatricians.

#### Timeliness

The median reporting time from date of delivery to date of report was 44 days (range 8–178) in 2013 for online reporting. Timeliness for the paper-based reporting could not be assessed because the date of reporting was not documented.

#### Stability

BDRI-C membership expanded from 15 members in 2001 to 46 members in 2013 in Chennai. In 2011, BDRI-C was registered as a member of ICBDSR. However, stability has been affected by inconsistent data reporting with different institutions reporting in different years.

## DISCUSSION

BDRI-C is a useful, simple, flexible, and timely passive surveillance system for birth defects among reporting institutions in Chennai. However, it is not representative of Chennai and improvements can be made in data quality, acceptability, and stability.

The registry is managed by a private organization (the MediScan systems), and its ability to capture data routinely, especially from the government institutions, is restricted at times. BDRI-C was initially established as a passive registry primarily to assess the prevalence of birth defects, but being a voluntary system of reporting birth defects, can help explain poor acceptance. This limitation is also reflected in the less than satisfactory representation of births captured by BDRI-C in Chennai. As a passive reporting system, it is crucial that member institutions are regularly encouraged and reminded to report data. For registered members in BDRI-C, the reporting has not been continuously monitored due to staff turnovers in BDRI-C coordinating team. Our study has several limitations. We were able to conduct interviews with only 6 out of 24 institutions that had ever reported data to BDRI-C. The accuracy of data reported by member institutions could not be verified, since medical records at member institutions were not accessible. Based on our findings, we provided suggestions to improve data quality by checking for completeness and timely requests for missing data including date of reporting. To improve representativeness so that data reflect the actual prevalence and epidemiological trends in Chennai, it might be helpful to contact registered institutions who have either stopped reporting data or never reported data to try to restart data reporting. Routine monitoring of data reporting by BDRI-C could be helpful.

Partnership with the state government of Tamil Nadu is important for birth defects efforts. Tamil Nadu is a state with good performances in reproductive and child health services, achieving 94% institutional deliveries and having schemes like the Muthulekshmi Reddy scheme (unconditional cash transfer) to promote institutional deliveries.^6,7^ This provides a valuable opportunity for government institutions to record birth defects for BDRI-C. Analyzing and publishing data in scientific journals will aid the government in planning and mapping the available government resources like the new Rashtriya Bal Suraksha Yojana program.

The evaluation and feedback provided an external assessment and offered suggestions for registry improvement. Presentation of these results to stakeholders at the World Health Organization (WHO) South East Asia Region meeting for birth defects in 2014 helped WHO establish the WHO SEARO Newborn and Birth Defects Database.^8,9^

## CONCLUSION

Birth defects surveillance is valuable in India, but greater visibility would help to ensure more recruitment of institutions and inspire the growth and utility of birth defects registries.

## Figures and Tables

**Table 1:** Birth Defects Registry of India-Chennai (BDRI-C) system attributes, definitions, and findings.

Level of usefulness/System Attribute*	Definition	Findings	Source of information
**Usefulness**	Ability of the system to contribute to the prevention and care of birth defects	BDRI-C is helpful in linking families impacted by birth defects to needed services; however, inconsistent reporting and varying data sources limit its ability to identify clusters and trends for birth defects in Chennai.	BDRI-C materials, such as reporting forms and documentation of data collection methods Key informant interviews
**Simplicity**	System’s method and ease of data collection	The system is simple. It has a 26 variable reporting form submitted via paper or online. Communication between reporting member institutions and BDRI-C staff are bidirectional, allowing data and aggregated reports and feedback.	BDRI-C materials, such as reporting forms and documentation of data collection methods Key informant interviews
**Flexibility**	Ability to modify reporting form and operations to meet changing needs	The system is flexible. Online reporting was introduced in 2009. Thrice, the reporting forms were changed to reduce or add more fields (online system and paper forms). 1) The number of reporting fields was reduced from 84 to 21 in 2007 following a general feedback that the number of reporting fields is burdensome for the members. 2) 4 new fields were added when BDRI-C became a member of the International Clearinghouse for Birth Defects Surveillance and Research in 2011. 3) An additional field, periconceptional folic acid consumption, was added as an optional field in 2012.	Reporting forms Key informant interviews
**Data quality**	Completeness and validity of the data recorded in the system	Phone number was the most frequently missing variable (34%, 32/95 in paper reporting forms and 31%, 17/55 in the online reports). In the online reports 14 records (25%, 14/55) were incomplete for address.	95 paper reporting forms from nine institutions and 55 online reports from eight institutions in 2013
**Representativeness**	Proportion of participating institutions reporting data to BDRI-C and the percent of births covered by these participating institutions	BDRI-C is not representative of Chennai because major government institutions and some private institutions are not registered and reporting their data. Among 698 maternity institutions in Chennai, 46 have registered in BDRI-C. Among registered institutions, 21 (46%), 14 (30%) and 17 (37%) reported data in 2011, 2012 and 2013 respectively (reporting institutions can vary by year). BDRI-C has data collected on 11,763 births from among the 81,209 births from April 2013 – March 2014. Therefore, the coverage of BDRI-C is 14%, which was a decrease in coverage from April 2011 to March 2012 (40%) and April 2012 to March 2013 (30%).	Reporting forms from member institutions to BDRI-C Birth data statistics from Greater Chennai Corporation
**Acceptability**	Willingness of persons and organizations to participate in the surveillance activities	Acceptability is low since very few maternity institutions in Chennai registered in BDRI-C and of those that registered, very few provided data 2011-2013. Staff turnovers at the reporting member institutions required on-going training and sensitization. Data review meetings in the institutions were perceived as academic and reported as being useful only for pediatricians.	BDRI-C materials Key informant interviews
**Timeliness**	Time from date of delivery to date of reporting to BDRI-C	The system is timely. The target time interval for reporting data to BDRI-C is 2 months. We found that the median reporting time was 44 (8–178) days in 2013. For the paper-based reporting, only date of birth is recorded in the form and not the date of report; therefore, timeliness could not be assessed in paper-based reporting.	55 online reports from eight institutions in the year 2013
**Stability**	Variations over time of the number of institutions participating in and reporting data to BDRI	In terms of membership, BDRI-C has been stable since 2001 and has expanded steadily from 15 members in Chennai in 2001 to 46 members in in 2013. BDRI-C has also expanded to capture data from other states in India. It has been registered as a member of the International Clearinghouse for Birth Defects Surveillance and Research since 2011. However, stability has been affected by inconsistent data reporting with different institutions reporting in different years.	BDRI-C materials Key informant interviews

* Sensitivity and predictive value positive could not be assessed in this evaluation.
